# Crystallographic and Biochemical Analysis of the Mouse Poly(ADP-Ribose) Glycohydrolase

**DOI:** 10.1371/journal.pone.0086010

**Published:** 2014-01-21

**Authors:** Zhizhi Wang, Jean-Philippe Gagné, Guy G. Poirier, Wenqing Xu

**Affiliations:** 1 Department of Biological Structure, University of Washington, Seattle, Washington, United States of America; 2 Axe Cancer, Centre de Recherche du Centre Hospitalier Universitaire de Québec, Faculty of Medicine, Laval University, Québec, Canada; Institute of Molecular and Cell Biology, Singapore

## Abstract

Protein poly(ADP-ribosyl)ation (PARylation) regulates a number of important cellular processes. Poly(ADP-ribose) glycohydrolase (PARG) is the primary enzyme responsible for hydrolyzing the poly(ADP-ribose) (PAR) polymer *in vivo*. Here we report crystal structures of the mouse PARG (mPARG) catalytic domain, its complexes with ADP-ribose (ADPr) and a PARG inhibitor ADP-HPD, as well as four PARG catalytic residues mutants. With these structures and biochemical analysis of 20 mPARG mutants, we provide a structural basis for understanding how the PAR polymer is recognized and hydrolyzed by mPARG. The structures and activity complementation experiment also suggest how the N-terminal flexible peptide preceding the PARG catalytic domain may regulate the enzymatic activity of PARG. This study contributes to our understanding of PARG catalytic and regulatory mechanisms as well as the rational design of PARG inhibitors.

## Introduction

Protein function and localization inside the cell are usually regulated by post-translational modifications (PTMs). Poly(ADP-ribosyl)ation (PARylation) is a reversible PTM that is involved in various cellular processes, including DNA repair, chromatin structure dynamics, gene transcription, poly(ADP-ribose) (PAR) dependent cell death (pathanatos) and PARylation dependent ubiquitination [1]–[5]. PARylation is catalyzed by a family of poly(ADP-ribose) polymerases (PARPs), which modify the target protein side chains by transferring the ADP-ribose (ADPr) moiety from nicotinamide adenine dinucleotide (NAD^+^). Reversal of PARylation is predominantly carried out by poly(ADP-ribose) glycohydrolase (PARG) in nucleus and cytosol, whereas ADP-ribosylhydrolase 3 (ARH3) may play a role in mitochondrial PAR degradation [6].

PARPs and PARG are present in all eukaryotic cell types except yeast [7]. A recent study showed that PARG homologues are also present in several species of bacteria [8]. There are several PARPs in humans, including PARP1, PARP2 and tankyrases, which share homology to the PARP1 catalytic domain. In contrast, there is only one *PARG* gene encoding for at least three different isoforms of PARG localizing in different cellular compartments. The 111 kDa full length PARG (hPARG_111_) localizes in the nucleus. Both 99 kDa hPARG_99_ and 102 kDa hPARG_102_ isoforms localize in the cytoplasm. While the N-terminal region is absent in some PARG splicing forms and predicted to be disordered (Fig. S1) [9], the conserved C-terminal 60 kD catalytic domain is fully active [10], [11].

PARG activity is essential for many cell types. Loss of PARG function in *Drosophila melanogaster* results in either lethality in the larval stage or progressive neurodegeneration, for survivors under certain conditions, with a reduced lifespan due to the excessive production of PAR in the central nervous system [12]. The PARG null mutation in mouse causes the lethal phenotype in early embryos [13]. The hypomorphic mutation of PARG (PARG_110_^−^/^−^) in mouse showed impaired DNA repair response with high genomic instability, including chromosome aberrations and a high frequency of sister chromatid exchange [14], [15].

It has been reported that vertebrate PARG possesses both exo-glycosidase and endo-glycosidase activities and therefore is able to hydrolyze ribose-ribose glycosidic bonds between ADP-ribose units at the terminus or within the PAR polymers [16], [17]. PARG hydrolyzes long polymers of ADP-ribose first. Branched and short PAR molecules are degraded slowly and with lower affinities by PARG (K_M_≈10 µM) than long and linear polymers (K_M_ = 0.1–0.4 µM) [18]–[20]. The PAR formed following the activation of PARP1 by DNA damage has a very short half-life [21]. It is mostly degraded by PARG only a few minutes after its synthesis. Thus PARG prevents the accumulation of highly PARylated proteins with long PAR modification in the nucleus and may also keep PARP1 active by removing PAR polymer which results from inhibitory PARP1 auto-PARylation.

Among proposed PARG inhibitors, adenosine 5′-diphosphate-(hydroxymethyl)-pyrrolidinediol (ADP-HPD), an analogue of ADPr, is probably the most potent and best studied one, with an IC_50_ of about 120 nM. ADP-HDP has been used for *in vitro* studies for PARG inhibition. However, it is not cell permeable and can be hydrolyzed by phosphodiesterases in the cell, which make it unsuitable for cell based studies. The lack of an ideal small compound inhibitor for PARG is still a major hurdle for function studies of PARG. Recently, inhibitors of PARG have been proposed as drug targets in pathophysiological conditions such as inflammation, ischemia, and stroke [22]–[25]. In addition, because PARG deficiency enhances cytotoxic sensitivity induced by chemotherapy agents [13], PARG inhibitors are potential anti-cancer drug sensitizers.

To understand how PARG catalyzes PAR degradation and how it is regulated, and to provide a structural basis for PARG inhibitor development, we have independently determined crystal structures of a mouse PARG fragment roughly corresponding to the fully-active 60 kD fragment, in apo-form, and in complexes with ADPr or a PARG inhibitor ADP-HPD. Our apo-mPARG structure was one of the first released eukaryotic PARG structures (PDB ID: 4FC2). During our manuscript preparation, crystal structures of the bacterial *T. curvata* PARG, and the PARG catalytic domains of protozoan *T. thermophila,* rat and human were reported [8], [26]–[29]. To further understand the catalytic and regulatory mechanisms of PARG, we have done a thorough mutagenesis analysis of mPARG and solved structures of mouse PARG in complex with various substrates and inhibitors. Our work revealed precisely how some of the PARG mutations (e.g. E748N, E749N) disrupt the PARG activity through significant conformational changes in the PARG active site. We also observed an unxpected binding site (outside of the catalytic cleft) for *iso*-ADP-ribose, which is probably the smallest PARG subtrate containing the α(1→2) ribose-ribose glycosidic bond, which may explain the processivity of PARG activity. Furthermore, through a complementation experiment, we show that the N-terminal regulatory fragment can activate *in trans* the inactive PARG fragment depleted with this segment. This suggests that, whereas the PARG activity can be inhibited by disrupting the docking of this segment to its PARG binding groove (via posttranslational modification or protein-proteins interactions), PARG can be reversibly activated once the disruptive factor is removed. Altogether, our crystallographic and biochemical studies provided further insights into the catalytic and regulatory mechanism of mamalian PARG.

## Results

### Crystal structures of the mouse PARG catalytic domain in apo- and liganded-states

PARG comprises an N-terminal regulatory/targeting domain and a C-terminal catalytic domain. The N-terminal region of mouse PARG (1–438) is absent in some PARG splicing forms, and is predicted to be disordered as shown by the metaPrDOS server (Figure S1) [30]. In comparison, the conserved C-terminal 60 kD catalytic domain is well-folded and fully active for PARG activity [11], [31]. We purified and crystallized the recombinant mouse PARG catalytic domain (residues 439–959) and determined the unliganded structure of mPARG(439–959) using Se-Met SAD method at 2.0 Å resolution (Table S1).

**Figure 1:**
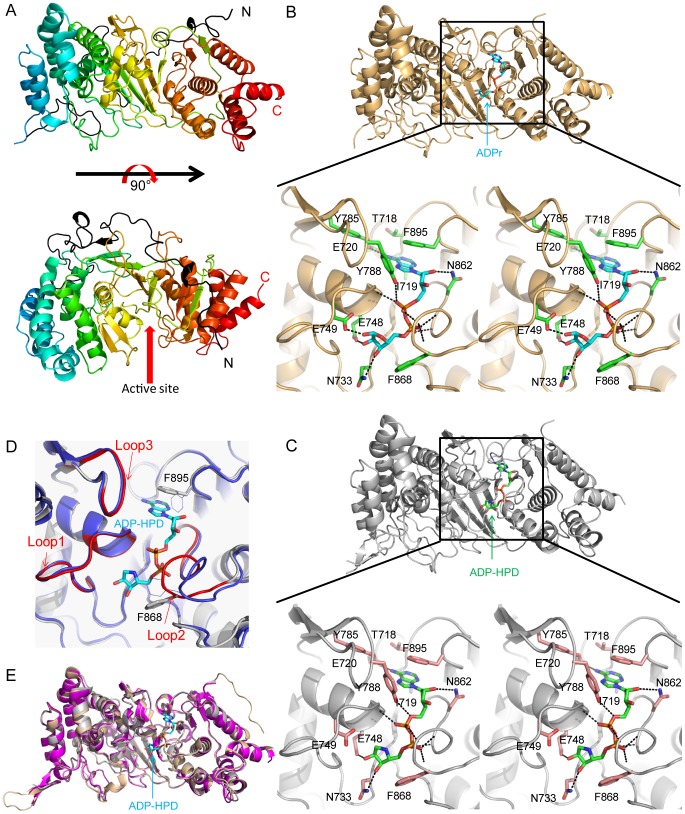
Mouse PARG catalytic domain apo- and ligand bound structures. (a) Overall structure of apo- mPARG(439–959). The protein is shown in rainbow and the N terminal MTS containing loop is in black. The cleft right in the middle is the active site. (b) ADPr bound mPARG structure. mPARG is shown in light orange and the ADPr is in cyan. Stereoview of key interactions involved in ADPr binding with the mPARG catalytic domain are shown in black dash lines. The key binding residues are highlighted in green sticks. (c) ADP-HPD bound mPARG structure. mPARG is shown in gray and the ADPr is in green. Stereoview of key interactions involved in ADP-HPD binding with the mPARG catalytic domain are shown in black dash lines. The key binding residues are highlighted in pink sticks. Aromatic rings of Tyr788 and Phe895 form perpendicular and parallel π stacking interactions with the adenine ring of ADPr or ADP-HPD, respectively. Tyr785 and Glu720 both form hydrogen bonds with the NH_2_ group of the adenine ring. Thr718 and Ile719 are in close contact with the N1 of the adenine ring. In addition, Asn862 forms a hydrogen bond to 2′-OH of the adenine-linked ribose. Tyr788 also forms a hydrogen bond with one of the phosphates. (d) Superposition of unliganded mPARG (blue) and ADP-HPD bound mPARG (grey) structures.Three key loops are highlighted in red in ADP-HPD bound structure. Loop 2 undergoes conformational change to tightly pack the ADP-HPD. Both side chains of Phe868 and Phe895 (highlighted in grey sticks) rotate to strongly interact with ADP-HPD. (e) Superposition of ADP-HPD bound vertebrate PARG catalytic domains. ADP-HPD bound mPARG is in grey, ADP-HPD bound rPARG (PDB: 3UEL) is in wheat and ADP-HPD bound hPARG (PDB: 4B1J) is in magenta. ADP-HPD is showed in cyan stick.

The mouse PARG catalytic domain has a bean-shaped structure, with the active site in a deep cleft in the middle on the abdominal side. A nine-strand mixed β sheet is sandwiched by two helical domains. The N-terminal helical domain has nine α helices, whereas the C-terminal helical domain has five. The very N-terminal segment of the mPARG catalytic domain contains the sixteen-residue putative mitochondrial targeting sequence (MTS, residues 454–469, MRKMPRCGIHLPSLRP) and binds to a groove on the side opposite to the active site (Figure 1A). The core structure of the mPARG catalytic domain also has an ADPr-binding macrodomain fold, despite missing the first β strand of the macrodomain (Figure S2).

There are three loops in the PARG catalytic cleft: the GGG-X_6-8_-QEE PARG signature catalytic loop (loop 1), the di-phosphate binding loop (loop 2) that is highly conserved among PARGs and other macrodomain structures [8], and the third loop (loop 3) from a β hairpin that is an additional segment in the macrodomain-like region (Figure S2). Tyr788, a residue previously identified to be important for the recognition of the PARG inhibitor ADP-HPD [32], is located at the tip residue in this β hairpin pointing into the cleft. The two consecutive Glu residues (E748 and E749) in the catalytic loop 1 are known to be key catalytic residues [33].

To explore how mPARG recognizes ADPr, its substrate/product unit, and ADP-HPD, a known PARG inhibitor, we tried to co-crystallize inhibitors with mPARG and to soak it into mPARG crystals. With the first crystal form that we solved mPARG structure (space P1), both methods failed, since the active site cleft is close to another copy of the mPARG molecule in the crystal lattice. Under a new crystallization condition, we obtained a second crystal form (space group P2_1_2_1_2) successfully soaked in ADPr and ADP-HPD, and solved the complex structures by molecular replacement (Table S1).

The overall structures of ADPr and ADP-HPD bound to PARG are similar to the unliganded structure. ADPr or ADP-HPD binds to the active site cleft of mPARG (Figure 1B, 1C). The signature catalytic loop (GGGVTGAGLVQEE) interacts with the ribose ring of ADPr or the pyrrolidine ring of the ADP-HPD. Residue Glu748 forms a hydrogen bond with the 2″-OH, while the key catalytic residue Glu749 side chain carboxyl group is very close to the 1′-OH of the ADPr or the C1″ of the ADP-HPD. Glu749 may work as a general acid to protonate the 2′-OH of adenine-linked ribose on the (n-1) ADPr. Residue Asn733 forms another hydrogen bond with 3″-OH to recognize the ligand. The glycine rich region may interact with the diphosphate group in the (n-1) ADPr, as suggested by the *T. thermophila* PARG crystal structure in complex with a short PAR polymer [29]. The second conserved glycine rich loop (GCGAFGGD) interacts with the diphosphate group of the ADPr or ADP-HPD. Residue Phe868 side chain is also in close contact with the ribose ring of ADPr or the pyrrolidine ring of ADP-HPD (Figure 1B, 1C).

On the adenine-linked ribose side, the adenine ring interacts extensively with mPARG (Figure 1B, 1C). All these interactions position the PAR polymer in the right orientation to be hydrolyzed by PARG. Upon binding, the second conserved glycine rich loop (GCGAFGGD) undergoes a major conformational change to tightly interact with the ADP-HPD (Figure 1D). The side chain dihedral angle of Phe868 rotates about 90° to form a close contact with the pyrrolidine ring. The side chain dihedral angle of Phe895 rotates about 120° to form parallel π stacking interactions with the adenine ring and subsequently close the deep pocket for the adenine ring (Figure 1D).

Comparison of ADP-HPD bound mouse PARG structure with other reported vertebrate PARG structures [27], [28] (rat PDB: 3UEL, r.m.s. deviation 0.310Å over all Cα atoms; human 4B1J, r.m.s. deviation 0.314Å over all Cα atoms) showed highly similar 3D structures of vertebrate PARG catalytic domain (Figure 1F). This is expected because of the high protein sequence similarity among these PARG catalytic domains.

### Mutagenesis analysis of PARG active site residues

**Figure 2:**
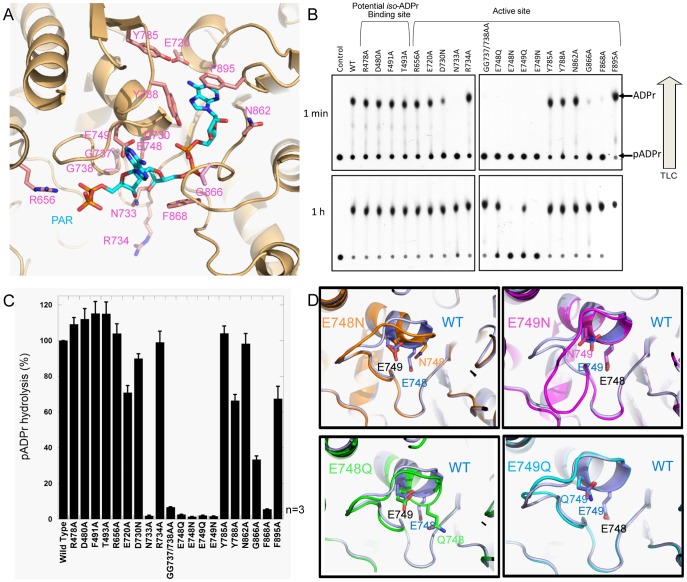
Mutagenesis analysis of mPARG active site residues. (a) The active site of ADPr bound mPARG structure is shown in light orange. The ligand PAR is modeled in based on superposition of the ADPr bound mPARG structure with PAR bound *T. thermophila* PARG structure (PDB: 4L2H). PAR is shown in cyan stick, and the residues we designed for mutagenesis study are shown in pink stick. (b) 1 min and 1 h PARG TLC assay for wt mPARG and mutants. R478A, D480A,F491A and T493A are the mutants for the potential *iso*-ADPr binding sites, and the rest are the mutants for the active site. (c) Quantified PARG activity by 1 min PARG TLC assay for wt mPARG and mutants. The activities are normalized to wt mPARG. Error bars represent standard deviation (n = 3). (d) The signature loops of the wt mPARG and E748 and E749 mutants. Wt mPARG in blue; E748N in orange; E749N in magenta; E748Q in green; E749Q in cyan. The side chains for residues 748 and 749 are shown in sticks.

To better understand PARG catalytic mechanism, we designed sixteen PARG mutations of residues in the catalytic cleft of mouse PARG (Figure 2A, S3). We used the PARG TLC assay to evaluate the relative activity of these mutants. E748 and E749 are the key catalytic residues in the signature loop [33]. Interestingly, while E748N and E749N mutants were completely dead, both E748Q and E749Q mutants still retained residual PARG activities (Figure 2B, 2C). Several other mutations, including N733A, GG737/738AA, G866A and F868A demonstrated lower PARG activities, while most of other mutants are still largely active. Among these mutation-sensitive residues, N733 directly recognizes the 3′-OH on the proximal ribose of (n) ADPr. G737 and G738 may be responsible to “read out” di-phosphate of the (n-1) ADPr. G886 possesses the unique torsional angle to interact with the di-phosphate of the (n) ADPr. F868 is important to interact hydrophobically with the proximal ribose of (n) APDr. The activities of each mutants were quantified and normalized to wild type PARG (Figure 2B, 2C). All these mutagenesis results are consistent with the structure.

### Crystal structure of inactive mPARG mutants

To understand what caused the activity difference between the mPARG E748N/Q and E749N/Q mutants, we solved the crystal structures of these mPARG mutants (Table S1). PARG E748Q and E749Q mutants have very similar conformation in the active site (Figure 2D), consistent with the fact that glutamine has the similar size of side chain with glutamate. In contrast, the signature loops in E748N and E749N mutants have significantly different conformation from the wild type PARG. In wild-type PARG structure, Cβ and Cγ of both E748 and E749 residues are semi-buried. Mutation to Asn, which has both Cγ and Nγ linked to Cβ causes a spatial collision. Therefore, the conformation changes in this catalytic loop completely abolish activity of mPARG E748N and E749N mutants.

### A potential secondary *iso*-ADPr binding site

To study the enzymatic mechanism of mPARG, we tried to soak the catalytic residue Glu748, Glu749 mutants E748N, E748Q, E749N and E749Q with the *iso*-ADPr [34], which may be the smallest PARG substrate containing the α(1→2) ribose-ribose glycosidic bond to be cleaved by PARG. In all above four PARG mutant crystal structures, we were not able to observe the electron density for *iso*-ADPr at the “active” site of mPARG (Table S1). For E748N and E749N mutants, this may result from the conformational change in the active site associated with the mutation (Figure 2D). For the E748Q and E749Q mutants, this may result from the partial catalytic activities of these mutations (Figure 2B) and/or the low affinity between PARG and *iso*-ADPr.

**Figure 3:**
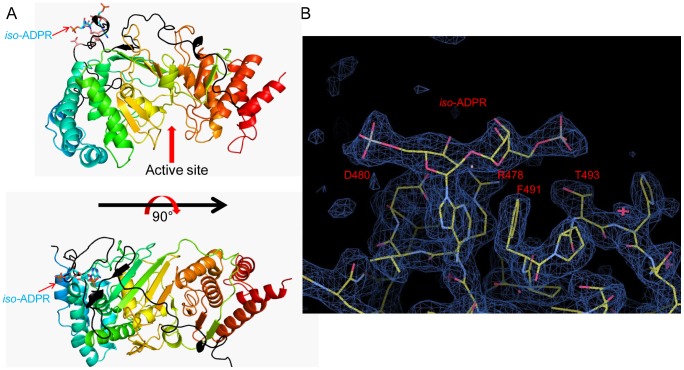
A potential secondary *iso*-ADPr binding site. (a) A possible secondary binding site. *iso*-ADPr is showed in cyan stick. The bound *iso*-ADPr is close to the Exon4+5 encoded region (highlighted in black). (b) The 2Fo-Fc simulated annealed omit map of the potential secondary *iso*-ADPr binding region, calculated using the CNS package and contoured at 1.5σ. The *iso*-ADPr was omitted and simulated annealing was performed to remove model bias prior to electron density calculation. It is also apparent that the *iso*-ADPr molecule in this position is not restricted by crystal packing.

Surprisingly, we repeatedly observed electron densities, in an unexpected position, which fit well with the chemical structure of *iso*-ADPr and refined well when *iso*-ADPr was built into the densities (Figures 3, S4). In these structures, *iso*-ADPr sits at the secondary binding site which is far away from the cleft (Figure 3A). This secondary binding site is at the mouse *PARG* exon 5 coded region, and is not formed due to crystal packing as it is far away from any PARG region involved in crystal packing (Figure 3B). There are four residues (R478, D480, F491 and T493) directly interacting with *iso*-ADPr (Figures 3B, S4). We purified these mutants and tested the activity by PARG TLC assay (Figures S3, 2B, 2C). However, we cannot detect any difference in PARG activity under our experimental condition. Whether this putative secondary *iso*-ADPr binding site is physiologically relevant in the context of complex PAR-protein assemblies in the cell awaits future investigation.

### A potential PARG regulatory mechanism: *in trans* complementation assay

**Figure 4:**
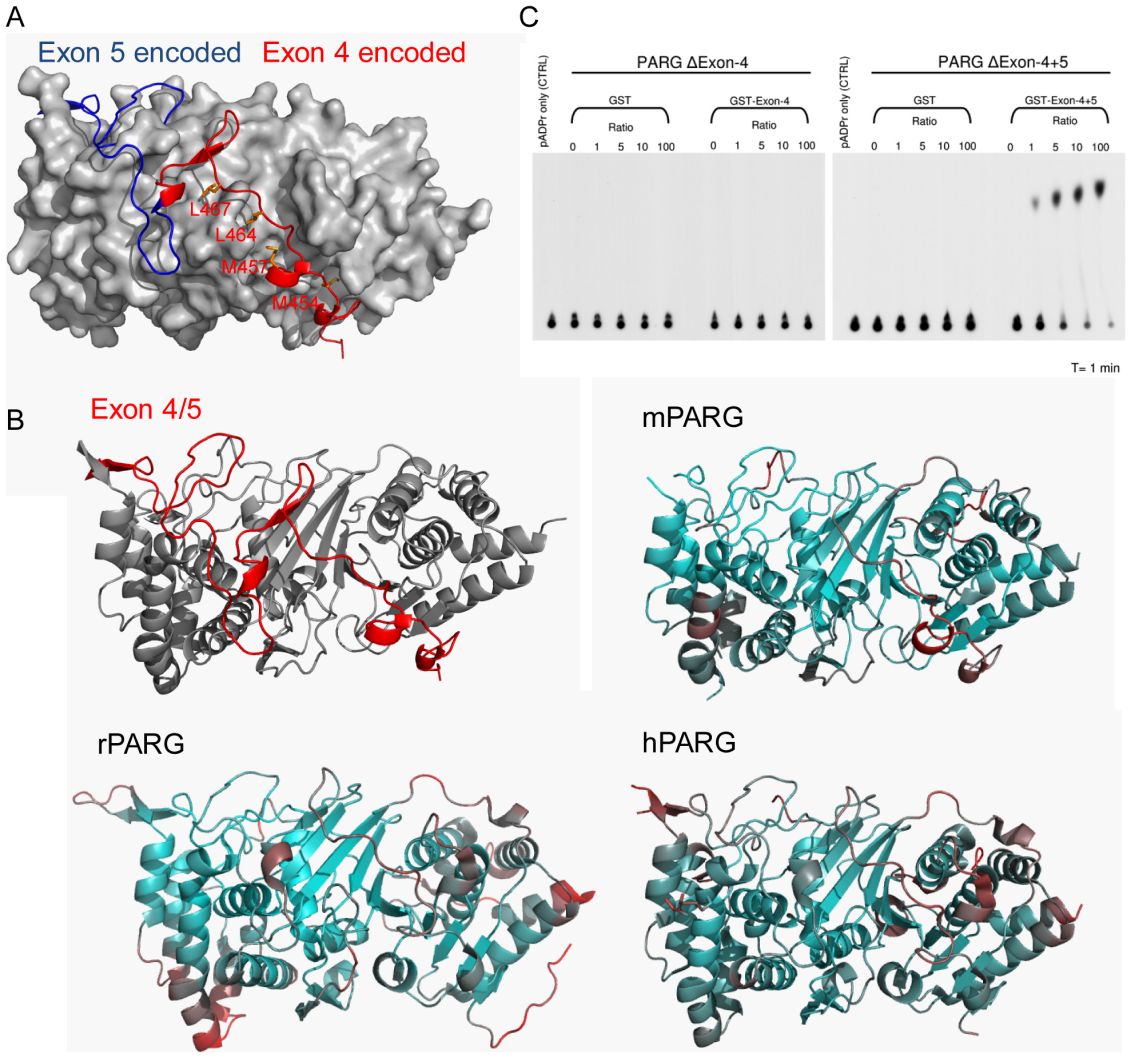
N-terminal exon4+5 encoded regulartory segment. (a) Exon 4+5 encoded segment docks on hydrophobic groove of the back side of mPARG catalytic domain. The core of mPARG is shown as grey surface. The exon 4 encoded region is shown in red, while the exon 5 encoded region is shown in blue. Met454, Met457, Leu464 and Leu467 are highlighted in orange sticks. (b) B factor spectrum of vertebrate PARG structures. The regions with low B factor are in cyan, and the ones with high B factor are in red. The exon4+5 encoded segments of mPARG, rPARG (PDB: 3UEK) and hPARG (PDB:4B1G) all have a relatively higher B factor than the core region of PARG catalytic domain. (c) *In trans* complementation PARG TLC assays with increasing peptide : PARG ratios.

Previous work has revealed the N-terminal MTS segment, which is encoded by the PARG exon 4, as a regulatory component of PARG activity [31]. This MTS is proposed to be the signal peptide to direct the import of PARG into mitochondria. Previous studies showed that MTS plays a crucial role in PARG activity, and the deletion or mutations of MTS result in the total or partial loss of PARG enzymatic activity [31]. In our mPARG structures, this MTS together with residues preceding it, has an extended conformation and wraps along the back side of the PARG catalytic domain (Figure 1A). The MTS docks in a hydrophobic groove on the back side of the β sheet, which is the opposite side from the active site (Figure 4A). Hydrophobic residues Met454, Met457, Leu464 and Leu467 on the MTS pack tightly with this hydrophobic groove (Figure 4A). This explains why the mutants of these leucine residues have no detectable enzymatic activity [31].

In addition, it was shown that deletion of the PARG segment encoded by exon 5 can also abolish PARG activity [6]. In our crystal structure, the PARG exon 4 encoded segment (residues 439–479), and exon 5 encoded segment (residues 480–519) together form an extended loop, and wrap around on the back side of PARG catalytic domain (Figure 1A). Most hydrophobic residues of exon 4 and 5 are buried towards the internal side of PARG structure in our crystal structure. It is interesting that the exon 4 and 5 encoded N-terminal segment has a relatively higher B factor than the core region of the PARG catalytic domain in the mPARG structures and other vertebrate PARG structures (Figure 4B). This indicates this N-terminal segment is structurally more dynamic than the core of PARG catalytic domain, a structural feature suitable for a regulatory role for PARG activity.

To further investigate how this N-terminal extended region affects the PARG activity, we purified GST-tagged exon 4 coded peptide (GST-Exon-4) and GST-tagged exons 4 and 5 coded peptide (GST-Exon-4+5), and PARG catalytic domain exon 4 coded region deleted (ΔExon-4) and exons 4 and 5 coded region deleted (ΔExon-4+5). We ran the PARG TLC assay to study whether these N-terminal peptides can restore the PARG activity of these inactive PARG fragments *in trans* (Figure 4C). Our data showed that while GST-Exon-4 could not rescue the PARG activity of ΔExon-4, GST-Exon-4+5 could restore the ΔExon-4+5 PARG activity *in trans* in a concentration dependent manner. This result suggests that docking or dislodge of the Exon(4+5) encoded segment may serve as a reversible PARG activity switch (see discussion).

## Discussion

### Implications in PARG catalytic mechanism

Based on the bacterial, protozoan, rat and human PARG structures, the mechanism of PARG catalysis was proposed to necessitate the binding of the terminal ADPr unit which in turn, positions the ribose-ribose O-glycosidic bond in direct hydrogen bonding contact with the last Glu residue of the signature catalytic loop (GGG-X_6-8_-QEE). A putative oxocarbenium intermediate is formed by the protonation of the (n-1) ADPr adenine-linked ribose 2′-OH leaving group through Glu. This positively charged oxocarbenium is stabilized by the proximal diphosphate group of bound ADPr. A water molecule is positioned to attack the oxocarbenium intermediate, which is activated through deprotonation by Glu. This results in the release of ADP-β-ribose and (n-1) PAR [8], [26]–[29]. Our PARG structure in complex with ADPr and ADP-HPD suggests that mouse PARG uses a very similar mechanism.

**Figure 5:**
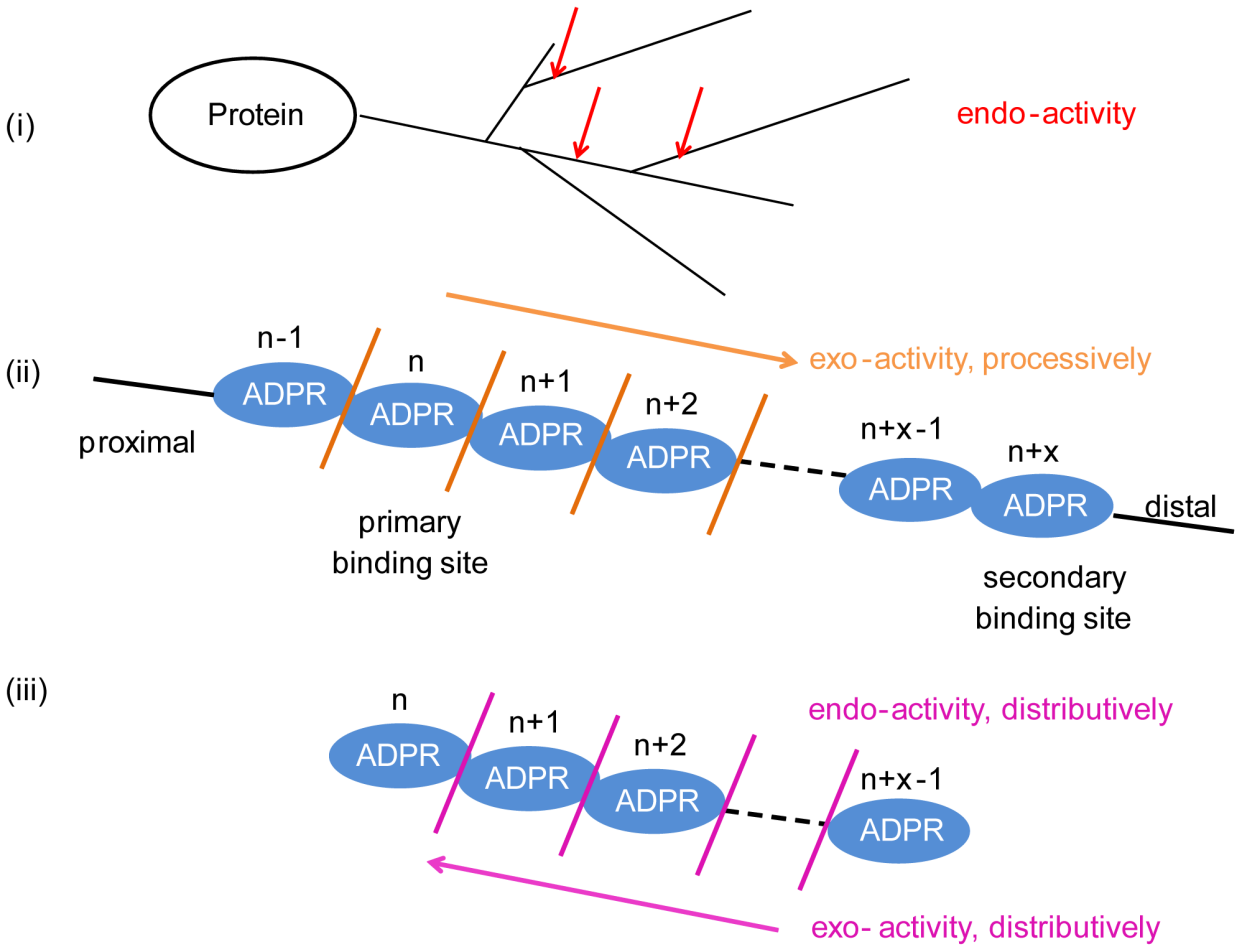
A model for different modes/stages of PAR degradation by PARG. Based on the data from this study and previous studies, we propose the catalytic mechanism of PAR degradation by PARG. In the early stage, PARG randomly recognizes the PAR between (n-1) and (n) ADPr, then hydrolyzes the glycosidic bond in-between (endo- activity). Because the (n-1) PAR polymer has lower binding affinity, it leaves PARG after the reaction. Long PAR polymers may have higher affinity with PARG than short PAR, due to the interaction between the (n+x) ADPr unit and the potential secondary *iso*-ADPr binding site. The (n+) PAR polymer stays with PARG after cleavage. Thereafter, PARG can slide along the (n+) PAR polymer to cleave ADPr units from proximal to distal end one by one (exo- activity). In the late stage, when the PAR polymer is not long enough, which result in the lower binding affinity with PARG (no secondary binding site), PARG can no longer processively degrade PAR polymers. Shorter PAR leaves PARG after every single cleavage, and is degraded by PARG distributively.

In contrast to bacterial PARG in which the 2′-OH of the adenine-linked ribose is buried, the same 2′-OH group in mouse PARG structure is exposed to the solvent, which would allow mPARG to bind (n+1) ADPr (Figure S5). Thus the bound ADPr unit in the vertebrate PARG active site can be either the terminal unit or an internal unit on PAR polymer, although the terminal unit may be favored [29]. A previous study showed that about 20% of the glycohydrolase activity of PARG proceed through endoglycosidic cleavage of PAR polymers [35]. The kinetic study of PARG showed there are three phases in PAR hydrolysis by PARG: (i) endoglycosidic cleavage, (ii) exoglycosidic, processive degradation, and (iii) distributive degradation (Figure 5) [36]. PARG also degrades longer PAR faster than shorter PAR [18]. It should be noted that all PARG crystal structures reported so far are consistent with the distributive degradation. That is, the PARG protein leaves the substrate PAR or PARylated protein after each cleavage, since the PARG protein predominantly interacts with the (n) ADPr while the cleavage happens between (n-1) ADPr and (n)ADPr. How does vertebrate PARG achieve the degradation processivity for long PAR polmers? We speculate that the second *iso*-ADPr binding site may play a role here. It is possible that a vertebrate PARG keeps binding to long PAR polymers during PAR degradation at the PARG active site and degrades PAR processively to small PAR chain until the PAR chain is too short to reach the putative second *iso*-ADPr site (corresponding to the phase ii). Then shorter PAR leaves PARG, and is degraded by PARG distributively (Figure 5). It has been technically very challenging to test if and how the second potential *iso*-ADPr binding site may contribute to PARG activity under physiologically conditions. It should be a topic of future investigations.

### Implication in PARG regulatory mechanisms

While the catalytic mechanism of vertebrate PARG is better studied, little is known about the regulatory mechanism of PARG activity. One structural component known to be crucial for PARG activity is the Exon 4–5 encoded region. Removal of either exon would abolish PARG activity [6], [31]. One explanation for the requirement of this region for PARG activity is to stabilize a “Tyr-clasp”, which forms a hydrogen bond between PARG Tyr788 (Tyr795 in human) and a phosphate group of (n) ADPr. However, this model cannot fully explain our and previous observations that mPARG Y788A and analogous PARG mutations are largely active (Figure 2B, 2C) [26], [32]. Alternatively, the removal or dislodging of this segment from the PARG surface opposite from the active site induces a major PARG conformational change that abolishes PARG activity. In this regard, it was shown that deletion of hPARG MTS (exon 4) region significantly increased α-helical content of the PARG catalytic domain [31].

The exon4+5 region contains ∼70 residues. The high conservation of this region among vertebrate PARGs (but absent in bacterial and protozoan PARGs) suggests an important regulatory role it may have for vertebrate PARG activities. Interestingly, in our structure and two other vertebrate PARGs, this exon 4 and 5 encoded region has relatively high B factors (thus more structurally dynamic) than the macro-domain like region of PARG (Figure 4B). We propose that the PARG activity can be regulated by either protein-protein interactions or posttranslational modifications that promotes the dislodging of the exon4+5 regulatory region from the PARG main body. This kind of dislodge may be initiated by change of local interactions (directly towards the exon 4–5 region) or through allosteric interactions in a site far from this region. The result we present in this work clearly demonstrates that this kind of PARG regulation is reversible. Once the regulatory factor (via protein-protein interaction or posttranslational modification) is removed, PARG can be reactivated by the incorporation of PARG 4+5 segment back to the PARG catalytic domain structure.

In summary, through the determination of high resolution structures of mPARG and PARG mutants, in both apo- and liganded states, and enzymatic assays of mPARG mutants, we provide a basis for understanding the catalytic mechanism for mouse PARG. Our structures and the activity complementation experiment also suggest a model for PARG regulation. All these works will be valuable for understanding the molecular mechanisms of PARG in cell regulation and for PARG inhibitor development.

## Materials and Methods

### Protein expression and purification

The gene fragment corresponding to mPARG catalytic domain (residues 439–959) was cloned into pGEX-4T1 with an N-terminal GST tag and a TEV cleavage site in-between. Native GST fusion protein was over-expressed in *E. coli* BL21 (DE3) cells (Novagen) grown in Luria broth media. Se-Met substituted GST-mPARG(439–959) was over-expressed in auto-induction media. Bacteria cell pellets were lysed by sonication. Both native and Se-Met GST fusion proteins were eluted from Glutathione Sepharose 4B beads. GST tag was removed by TEV at 4°C overnight. Then the proteins were further purified by an anion exchange column, and finally purified by a Superdex 200 column on FPLC (GE Healthcare). The peak fractions were pooled, and concentrated to ∼5 mg/ml in a buffer containing 10 mM Tris HCl pH 8.5, 100 mM NaCl, 2 mM DTT. The mutants of mPARG(439–959) were cloned by site-directed mutagenesis. The mutant proteins were expressed and purified using the same methods as for the wild type protein.

### Crystallization and structure determination

The hanging-drop vapor diffusion method for crystallization was used to prepare crystals of the Se-Met mPARG(439–959). To obtain protein crystals for structural studies of unliganded and mutants mPARG E748N, E749N, E748Q and E749Q, 1 µL of protein sample (5 mg/mL) was mixed with 1 µL of well solution containing 20% PEG3350, 0.2 M (NH_4_)_2_SO_4_ at 4°C. The best crystals were obtained by further micro-seeding in 14% PEG3350, 0.2 M (NH_4_)_2_SO_4_. Thick plate-shaped crystals usually appeared in one day at 4°C after seeding and grew to their full sizes in three days. The crystals were frozen by liquid nitrogen in the cryo solution containing 10% glycerol and 20% PEG3350. For the crystals of ADPr and ADP-HPD bound structures, 1 µL of protein sample with 25% glycerol was mixed with 1 µL of well solution containing 0.22 M KI, 20% PEG3350, 10 mM DTT at room temperature. The crystals grew to full size in 2–3 days, and were soaked with 1 mM ADPr or ADP-HPD overnight at room temperature. Then they were frozen by liquid nitrogen in cryo solution containing 10% glycerol and 20% PEG3350.

Screening and data collection were performed at the Advanced Light Source (ALS), beamlines 8.2.1 and 8.2.2 at wavelength 0.9793 Å. All diffraction data were processed by HKL2000 [37]. The unliganded structure was determined by single-wavelength anomalous dispersion (SAD) using one data set collected at wavelength 0.9793 Å, which was also used for refinement. The selenium sites and the initial phases were determined by PHENIX [38]. Thirty-six selenium sites were found in one asymmetric unit, and the experimental electron density map clearly showed the presence of four molecules of mPARG(439–959) in one asymmetric unit. The initial phases for ADPr and ADP-HPD bound mPARG(439–959) and E748N, E749N, E748Q and E749Q mutants were determined by molecular replacement in Phaser [39]. All models were improved using iterative cycles of manual rebuilding with the program COOT [40] and refinement with Refmac5 of the CCP4 6.1.2 program suite [41].

### Synthesis of ^32^P-labeled automodified PARP-1

^32^P-labeled automodified PARP-1 was synthesized essentially as described by Ménard and Poirier [42] in a total reaction volume of 900 µl [100 mM Tris-HCl pH 8.0, 10 mM MgCl_2_, 8 mM dithiothreitol (DTT), 10% (v/v) glycerol, 25 µg/ml calf thymus activated DNA, 1 mM NAD+ and 125 µCi of ^32^P-NAD^+^]. Ethanol was added to this preparation dropwise at 10% (v/v) final concentration, with constant mixing, and the reaction mixture was incubated for 3 min at 30°C. The reaction was started by adding 20 units of PARP-1 purified up to the DNA–cellulose step (600 units/mg of protein) as described by Zahradka and Ebisuzaki [43]. After 30 min at 30°C, during which time the enzyme was modified by covalent linkage of pADPr chains, 100 µl of 3 M sodium acetate (pH 5.2) and 700 µl of isopropanol were added as described by Brochu *et al.* [35]. The reaction mixture was kept on ice for 30 min and then centrifuged at 10 000×g for 10 min at 4°C. The pellet was washed 5 times with ice-cold 80% (v/v) ethanol and resuspended in 450 µl of water. Calculating from the radioactivity count before and after synthesis, the final pADPr concentration was 200 µM.

### PARG activity assays

PARG assays were performed in a final volume of 20 µl containing 20 mM potassium phosphate (pH 7.2), 50 mM KCl, 0.1 mg/ml BSA, 0.1% Triton X-100, 10 mM DTT and 20 µM of ^32^P-labeled automodified PARP-1. pADPr hydrolysis was started by the addition of PARG mutants to a final concentration of 0.1 µM. Samples were incubated at 30°C for the indicated times. PARG activity was measured by analysing the production of ADP-ribose monomers from automodified PARP-1. PEI-F (polyethyleneimine F) cellulose (Macherey-Nagel) TLC developed in 0.3 M LiCl and 0.9 M acetic acid according to Ménard and Poirier [42] was used to separate pADPr from ADP-ribose monomers generated by PARG. TLC plates were air dried and subjected to phosphor screen-based autoradiography on a Storm 8600 imager (Amersham).

### *In trans* complementation assay

GST, GST-Exon-4 or GST-Exon-4+5 were pre-incubated with PARG ΔExon-4 or ΔExon-4+5 at different molar ratio. Then the PARG activity assay was performed as above, and the result was analyzed by TLC.

### SDS-PAGE, Sypro staining

Proteins were resolved using 4–12% Criterion™ XT Bis-Tris gradient gel (Bio-Rad) and stained with Sypro Ruby (Bio-Rad) according to the manufacturer's instructions. Images were acquired using the Geliance CCD-based bioimaging system (PerkinElmer).

### Accession codes

Protein Data Bank: Diffraction data and coordinates of mouse PARG catalytic domain are deposited under accession codes 4FC2, 4NA0, 4NA4, 4NA5, 4NA6, 4N9Y and 4N9Z, respectively.

## Supporting Information

Figure S1 **The disorder prediction for mouse PARG from metaPrDOS server.** The X-axis corresponds to mouse PARG residue numbers 1–969. The Y axis is the disorder tendency for each residue. The blue curve is the average result from six different programs/servers, as summarized by the metaPRDOS server. Higher values indicate higher disorder propensity. It indicates the N-terminal regulatory domain of mPARG (1–438) is disordered, whereas the mPARG(439–959) protein that was used for crystallization trials was predicted to be well-folded.
(PDF)

Figure S2 **The core structure of mPARG has a macrodomain-like fold.** The macrodomain-like region is highlighted in pink. mPARG has more delicate structure than macrodomain, including the N-terminal extended loop, seven more helices in the N-terminal helix bundle, two more helices in the C-terminal helix bundle, and three more N-terminal β strands (all highlighted in green). In addition, mPARG has an additional segment that contains the “Tyr” clasp (highlighted in red) within the macrodomain-like region.
(PDF)

Figure S3 **Coomassie Blue Stained SDS-PAGE for the purified recombinant mPARG(439–959) wild type and mutants.** The red asterisk indicates the expected position for mPARG(439–959).
(PDF)

Figure S4 **A potential secondary** ***iso*** **-ADPr binding site.** **(A)** Stereoview of the *iso*-ADPr binding site. R478, D480, F491 and T493 are highlighted in pink sticks. These residues are highly conserved in vertebrate PARGs. *Fo - Fc* difference density (grey mesh) is calculated when *iso*-ADPr is omitted (contoured at 2.5 σ). **(B, C)** *iso*-ADPr is also observed in both E748Q and E749Q mutants structures at the same site. E748Q is in p2_1_2_1_2 space group **(B)**, and E749Q is in p2_1_ space group **(C)**. *Fo - Fc* difference density (grey mesh) is calculated when *iso*-ADPr is omitted (contoured at 2.5 σ).
(PDF)

Figure S5 **The 2′-OH group of the adenine-linked ribose is exposed to solvent.** The surface of the mPARG is shown as grey. ADPr analog ADP-HPD is highlighted as sticks. Unlike bacterial PARG, mPARG does not block the 2′-OH of the adenine-linked ribose. This allows the binding of (n+1) ADPr unit. This structure feature supports that mPARG has both exo- and endo-glycohydrolase activity.
(PDF)

Table S1 **Statistics for data collection and structure refinement of mouse PARG(439–959) crystals.**
(PDF)
