# Supplementary material for: Crystallographic and Biochemical Analysis of the Mouse Poly(ADP-Ribose) Glycohydrolase
Source: PLoS One. 2014 Jan 21;9(1):e86010. doi: 10.1371/journal.pone.0086010 (PMC3897571; doi:10.1371/journal.pone.0086010)
Supplement: Figure S1 — The disorder prediction for mouse PARG from metaPrDOS server. The X-axis corresponds to mouse PARG residue numbers 1–969. The Y axis is the disorder tendency for each residue. The blue curve is the average result from six different programs/servers, as summarized by the metaPRDOS server. Higher values indicate higher disorder propensity. It indicates the N-terminal regulatory domain of mPARG (1–438) is disordered, whereas the mPARG(439–959) protein that was used for crystallization trials was predicted to be well-folded. (PDF) [file pone.0086010.s001.pdf]

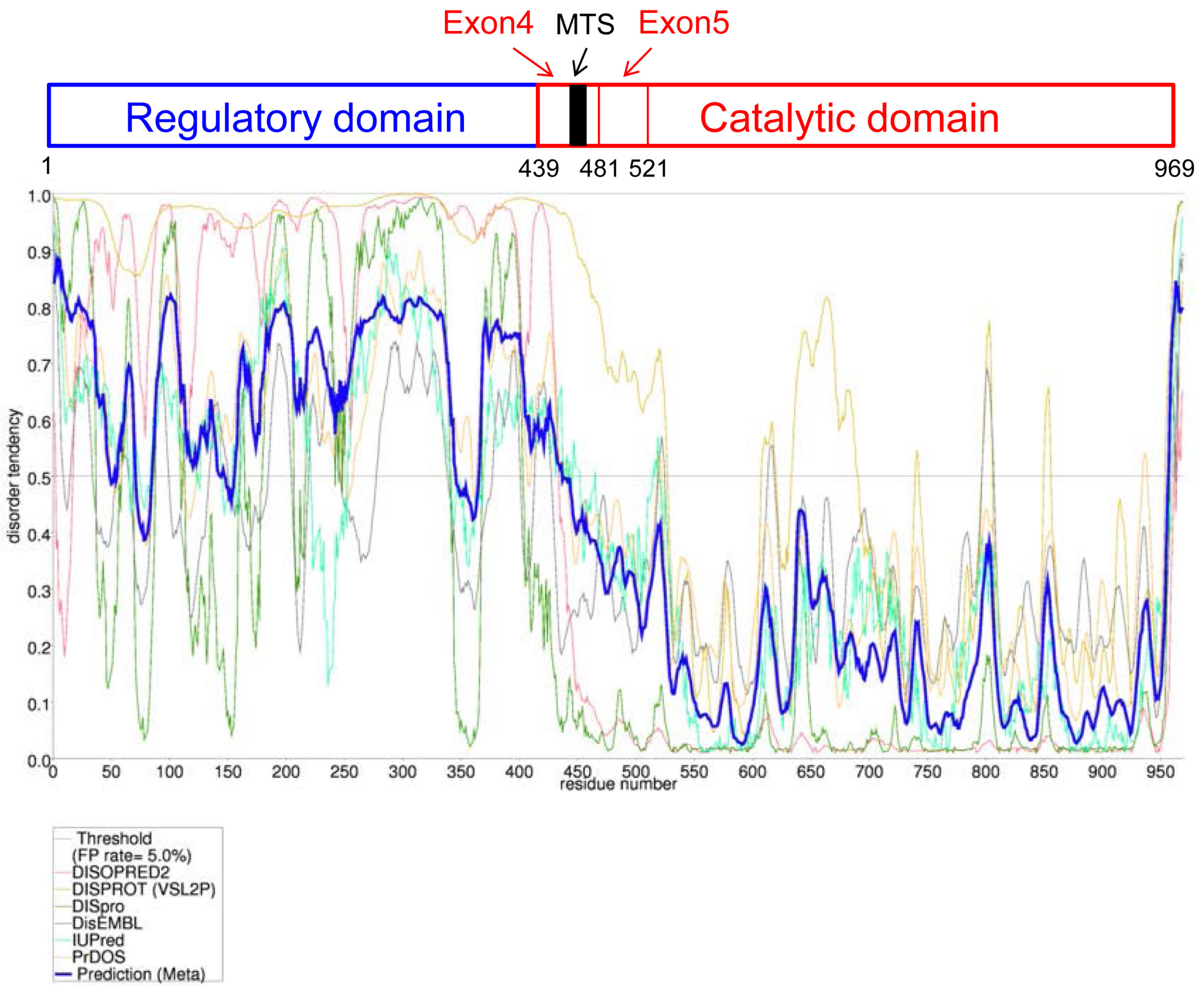
Exon4
MTS
Exon5
Regulatory domain
Catalytic domain
1
439
481
521
969
disorder tendency
residue number
Threshold
(FP rate= 5.0%)
DISOPRED2
DISPROT (VSL2P)
DISpro
DisEMBL
IUPred
PrDOS
Prediction (Meta)
