# Supplementary figures and images for: Crystallographic and Biochemical Analysis of the Mouse Poly(ADP-Ribose) Glycohydrolase
Source: PLoS One. 2014 Jan 21;9(1):e86010. doi: 10.1371/journal.pone.0086010 (PMC3897571; doi:10.1371/journal.pone.0086010)

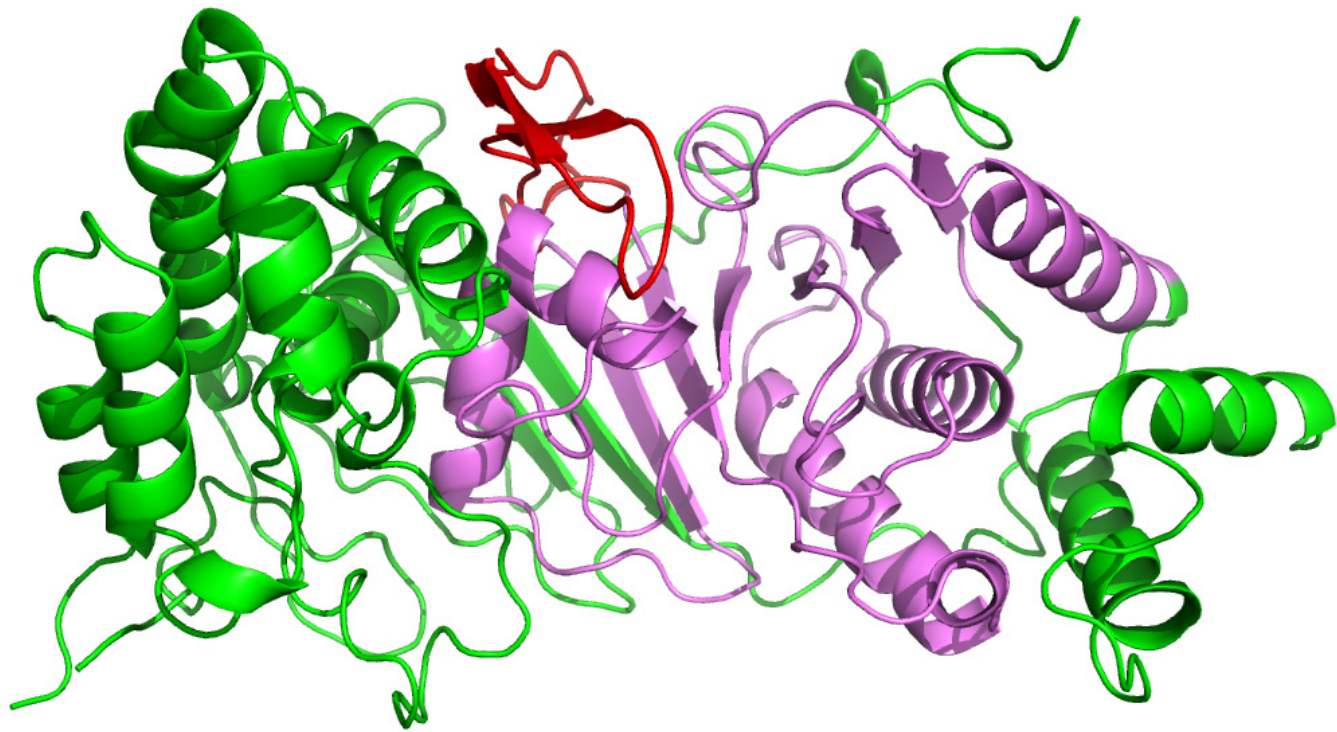

Supplement: Figure S2 — The core structure of mPARG has a macrodomain-like fold. The macrodomain-like region is highlighted in pink. mPARG has more delicate structure than macrodomain, including the N-terminal extended loop, seven more helices in the N-terminal helix bundle, two more helices in the C-terminal helix bundle, and three more N-terminal β strands (all highlighted in green). In addition, mPARG has an additional segment that contains the “Tyr” clasp (highlighted in red) within the macrodomain-like region. (PDF) [file pone.0086010.s002.pdf]

**A**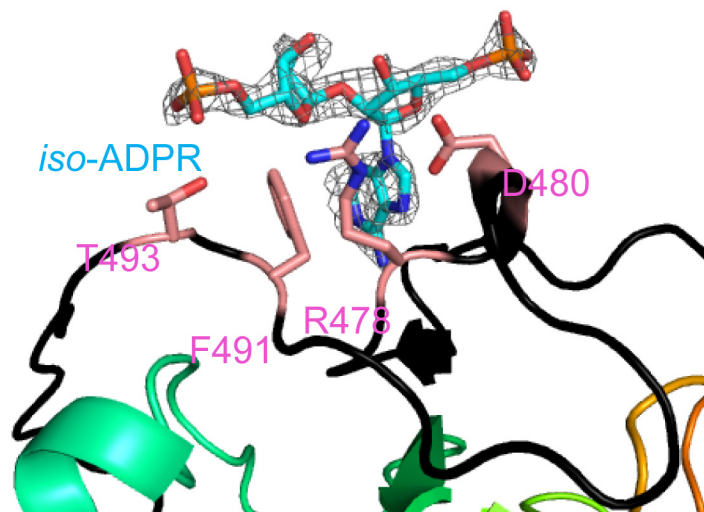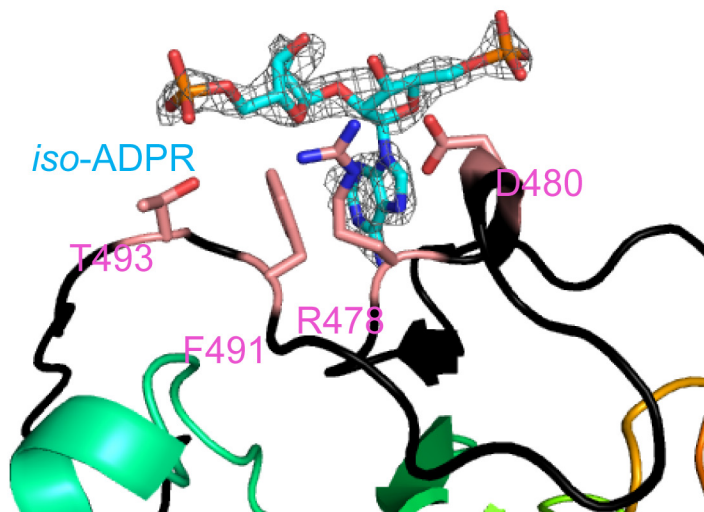**B**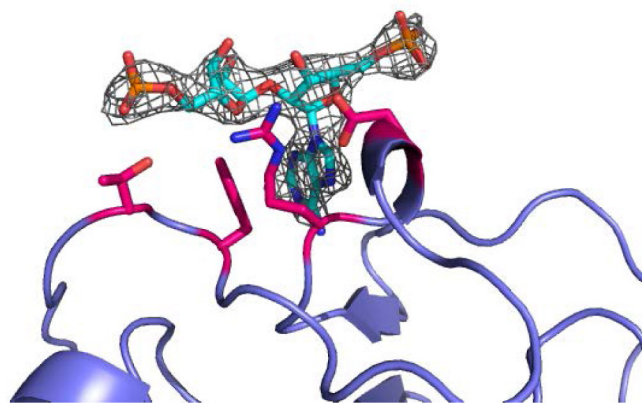**C**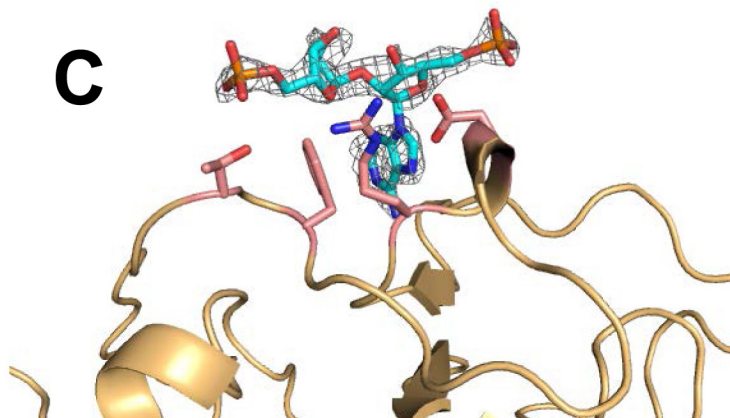

Supplement: Figure S4 — A potential secondary iso -ADPr binding site. (A) Stereoview of the iso-ADPr binding site. R478, D480, F491 and T493 are highlighted in pink sticks. These residues are highly conserved in vertebrate PARGs. Fo - Fc difference density (grey mesh) is calculated when iso-ADPr is omitted (contoured at 2.5 σ). (B, C) iso-ADPr is also observed in both E748Q and E749Q mutants structures at the same site. E748Q is in p21212 space group (B), and E749Q is in p21 space group (C). Fo - Fc difference density (grey mesh) is calculated when iso-ADPr is omitted (contoured at 2.5 σ). (PDF) [file pone.0086010.s004.pdf]

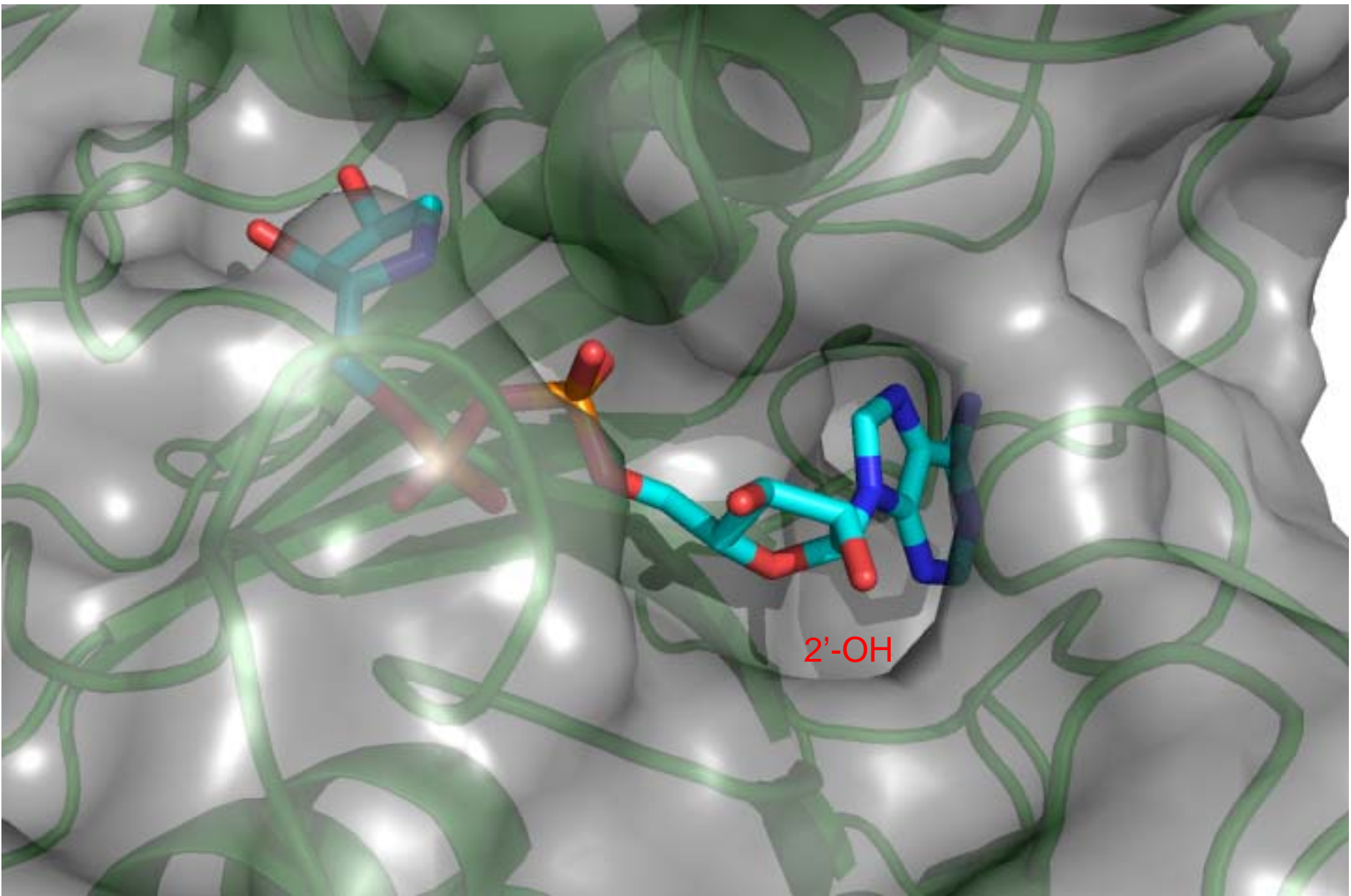

Supplement: Figure S5 — The 2′-OH group of the adenine-linked ribose is exposed to solvent. The surface of the mPARG is shown as grey. ADPr analog ADP-HPD is highlighted as sticks. Unlike bacterial PARG, mPARG does not block the 2′-OH of the adenine-linked ribose. This allows the binding of (n+1) ADPr unit. This structure feature supports that mPARG has both exo- and endo-glycohydrolase activity. (PDF) [file pone.0086010.s005.pdf]
