# Supplementary material for: Crystallographic and Biochemical Analysis of the Mouse Poly(ADP-Ribose) Glycohydrolase
Source: PLoS One. 2014 Jan 21;9(1):e86010. doi: 10.1371/journal.pone.0086010 (PMC3897571; doi:10.1371/journal.pone.0086010)
Supplement: Figure S3 — Coomassie Blue Stained SDS-PAGE for the purified recombinant mPARG(439–959) wild type and mutants. The red asterisk indicates the expected position for mPARG(439–959). (PDF) [file pone.0086010.s003.pdf]

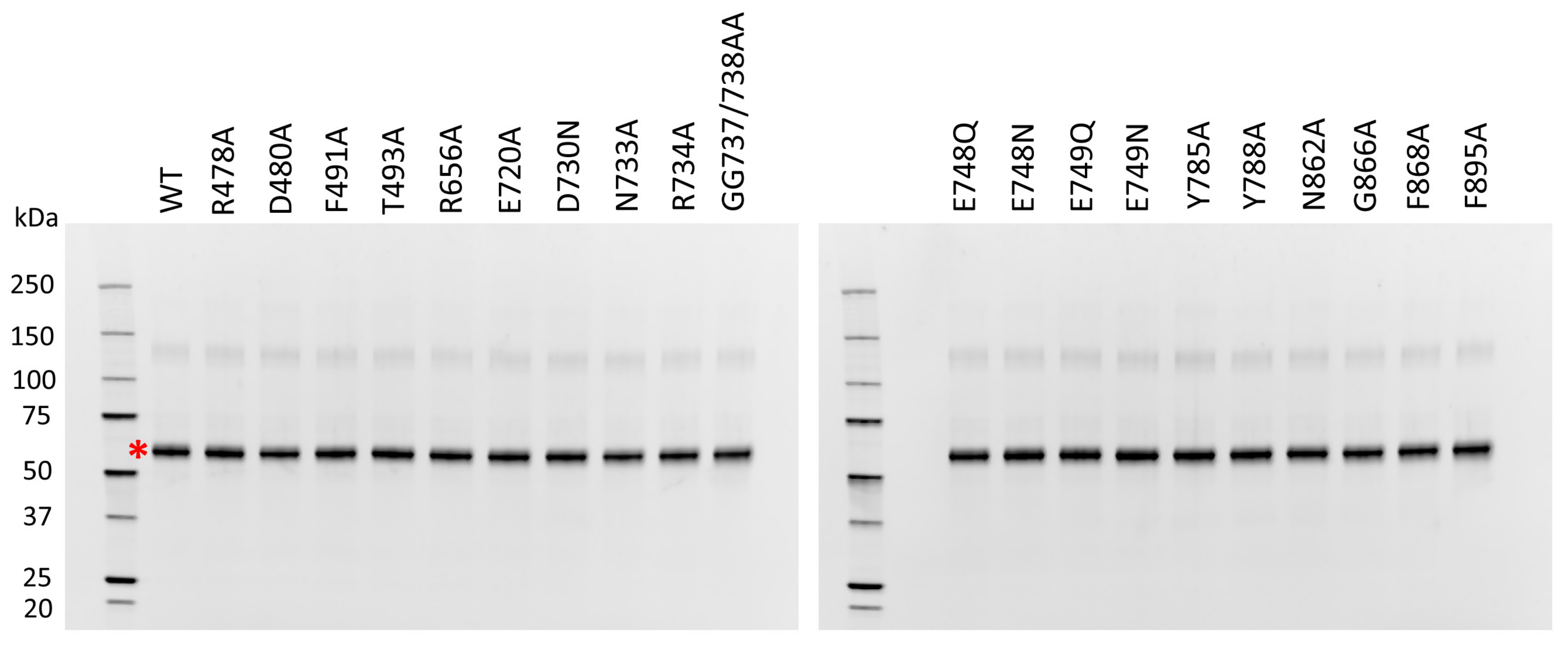

kDa
250
150
100
75
50
37
25
20
*
WT
R478A
D480A
F491A
T493A
R656A
E720A
D730N
N733A
R734A
GG737/738AA
E748Q
E748N
E749Q
E749N
Y785A
Y788A
N862A
G866A
F868A
F895A
