# Supplementary material for: Crystallographic and Biochemical Analysis of the Mouse Poly(ADP-Ribose) Glycohydrolase
Source: PLoS One. 2014 Jan 21;9(1):e86010. doi: 10.1371/journal.pone.0086010 (PMC3897571; doi:10.1371/journal.pone.0086010)
Supplement: Table S1 — Statistics for data collection and structure refinement of mouse PARG(439–959) crystals. (PDF) [file pone.0086010.s006.pdf]

Table S1. Statistics for data collection and structure refinement of mouse PARG(439-959) crystals.

	**SeMet SAD WT**	**ADPr bound**	**ADP-HPD bound**	**E748N**	**E749N**	**E748Q**	**E749Q**
**PDB code**	**4FC2**	**4NA0**	**4NA4**	**4NA5**	**4NA6**	**4N9Y**	**4N9Z**
**Data collection**							
Space group	$P1$	$P2_12_12$	$P2_12_12$	$P2_12_12$	$P2_1$	$P2_12_12$	$P2_1$
Cell dimensions							
a, *b*, *c* (Å)	67.14, 90.4, 104.7	188.30, 55.67, 166.15	188.95, 55.57, 165.99	67.09, 91.29, 103.50	91.60, 66.98, 101.55	67.24, 91.10, 102.76	66.92, 91.28, 102.52
α, β, γ (°)	81.64, 88.41, 89.36	90, 90, 90	90, 90, 90	90, 90, 90	90, 98.00, 90	90, 90, 90	90, 91.20, 90
Resolution (Å)	50.0-2.00 (2.06-2.00) *	50.0-2.50 (2.57-2.50) *	40.0-2.60 (2.68-2.60) *	50.0-2.00 (2.06-2.00) *	50.0-2.50 (2.57-2.50) *	50.0-2.30 (2.37-2.30) *	30.0-1.90 (1.96-1.90) *
*R*sym(%)	12.6 (50.4)	10.6 (73.5)	8.6 (56.0)	13.0 (67.4)	9.1 (46.8)	11.4 (51.4)	11.0 (33.4)
$I/\sigma I$	15.5 (1.9)	43.1 (3.7)	25.4 (2.3)	15.9 (1.8)	10.8 (1.7)	13.7 (2.2)	13.4 (3.9)
Completeness (%)	97.0 (84.9)	98.8 (96.6)	99.8 (99.3)	99.3 (92.4)	94.5 (59.8)	98.1 (90.7)	99.1 (97.8)
Redundancy	3.4 (3.0)	13.6 (12.5)	5.2 (4.8)	8.8 (4.2)	2.5 (2.0)	5.7 (3.5)	4.5 (4.3)
**Refinement**							
Resolution (Å)	50..0-2.00	50.0-2.50	40.0-2.60	50.0-2.00	50.0-2.50	50.0-2.30	30.0-1.90
No. reflections	151619	57525	51879	41102	38579	26481	90337
R_{work}/ R_{free}	17.0 / 21.2	29.8 / 34.1 [†]	23.5 / 27.7	18.0 / 21.4	21.6 / 25.5	19.7 / 24.0	19.0 / 23.0
No. atoms							
Protein	16481	12223	12219	4097	8210	4117	8251
Ligand	0	108	105	0	0	36 [#]	72 [#]
Water/ion	1623	0	97	430	40	254	1017
Average B-factors							
Protein	28.1	21.9	31.2	11.1	20.4	35.7	18.1
Ligand	N/A	42.8	47.3	N/A	N/A	53.6 [#]	40.2 [#]
Water/ion	29.8	N/A	50.5	16.5	50.4	37.4	25.0
R.m.s deviations							
Bond lengths (Å)	0.009	0.007	0.005	0.009	0.006	0.007	0.011
Bond angles (°)	1.135	1.275	1.069	1.141	0.989	1.169	1.287

For each dataset, a single crystal was used for data collection.
*Highest resolution shell is shown in parenthesis.
[†]High *R*work/*R*free are due to the pseudo-translation in this crystal form.
[#]The ligand for these two structures is *i*so-ADPr.
